# Nutritional Treatment of Patients with Colorectal Cancer

**DOI:** 10.3390/ijerph19116881

**Published:** 2022-06-04

**Authors:** Agata Lewandowska, Urszula Religioni, Aleksandra Czerw, Andrzej Deptała, Beata Karakiewicz, Olga Partyka, Monika Pajewska, Katarzyna Sygit, Elżbieta Cipora, Kamila Kmieć, Anna Augustynowicz, Dominika Mękal, Michał Waszkiewicz, Agnieszka Barańska, Daniela Mináriková, Peter Minárik, Piotr Merks

**Affiliations:** 1 Nutritional Treatment Clinic, Institute of Hematology and Transfusion Medicine, 00-791 Warsaw, Poland; agatkowska@tlen.pl; 2 School of Public Health, Center of Postgraduate Medical Education of Warsaw, 01-826 Warsaw, Poland; urszula.religioni@gmail.com (U.R.); aaugustynowicz@poczta.onet.pl (A.A.); michal.waszkiewicz@cmkp.edu.pl (M.W.); 3 Department of Health Economics and Medical Law, Medical University of Warsaw, 02-091 Warsaw, Poland; 4 Department of Economic and System Analyses, National Institute of Public Health NIH-National Research Institute, 00-791 Warsaw, Poland; opartyka@pzh.gov.pl (O.P.); mpajewska@pzh.gov.pl (M.P.); 5 Department of Cancer Prevention, Medical University of Warsaw, 02-091 Warsaw, Poland; andrzej.deptala@wum.edu.pl (A.D.); dmekal@wum.edu.pl (D.M.); 6 Subdepartment of Social Medicine and Public Health, Department of Social Medicine, Pomeranian Medical University in Szczecin, 70-103 Szczecin, Poland; beata.karakiewicz@pum.edu.pl; 7 Faculty of Health Sciences, Calisia University, 62-800 Kalisz, Poland; ksygit@poczta.onet.pl (K.S.); k.kmiec@akademia.kalisz.pl (K.K.); 8 Faculty of Medical Science, Jan Grodek State University in Sanok, 38-500 Sanok, Poland; ecipora@up-sanok.edu.pl; 9 Department of Medical Informatics and Statistics with E-Learning Lab, Medical University of Lublin, 20-090 Lublin, Poland; agnieszkabaranska@umlub.pl; 10 Faculty of Pharmacy, Comenius University in Bratislava, 814 99 Bratislava, Slovakia; minarikova@fpharm.uniba.sk; 11 Institute for Prevention and Intervention, St. Elizabeth University of Health and Social Work, 811 02 Bratisla-va, Slovakia; peterminarik57@gmail.com; 12 Biomedical Research Center of the Slovak Academy of Sciences, 845 04 Bratislava, Slovakia; 13 Department of Pharmacology and Clinical Pharmacology, Faculty of Medicine, Collegium Medicum, Cardi-nal Stefan Wyszyński University, 01-815 Warsaw, Poland; piotrmerks@googlemail.com

**Keywords:** cancer, colorectal cancer, neoplasms, nutritional deficiency, malnutrition

## Abstract

Colorectal cancer is one of the most common cancers in Europe and the world. Cancer treatments have side effects and cause significant deterioration of the patient’s nutritional status. Patient malnutrition may worsen the health condition and prevent the deliberate effects of the therapy. The aim of this review was to describe the available data about clinical nutrition in colorectal cancer patients. A large proportion of colorectal cancer patients suffer from malnutrition, which negatively affects the survival prognosis, quality of life, and oncological therapy. Therefore, monitoring nutritional status during the treatment is essential and can be used to arrange proper nutritional therapy to enhance patient responses, prevent side effects, and shorten recovery time. The principles of nutrition during anticancer therapy should mainly consider light and low-fat foods, the exclusion of lactose and gluten-containing foods in certain cases, or the introduction of special dietary products such as oral nutrition supplements and it should be tailored to patients’ individual needs.

## 1. Introduction

Colorectal cancer is one of the most common cancers in Europe and the world [1,2]. The main risk factors predisposing individuals to colorectal cancer include genetic factors, as well as lifestyle factors such as poor diet and obesity. The primary treatment for neither advanced nor metastatic colorectal cancer is a radical surgical resection. Depending on the stage of colorectal cancer, other treatments include chemotherapy, radiotherapy and molecular targeted therapy [3]. It is worth underlining that cancer treatment causes side effects that affect the nutrition status. Therefore, it is necessary to follow special nutrition rules in patients with colorectal cancer. Nutrition should be adjusted to the condition and individual needs of patients. It has been proven that cancer is inseparable from the reduction or exhaustion of body protein reserves and many vitamins (such as vitamin D, vitamin C, vitamin E, folic acid) and minerals (e.g., selenium, iron, zinc) [4,5,6]. Mobilization of body protein reserves occurs through intensification of selected metabolic pathways: anaerobic glycolysis, gluconeogenesis and lipolysis increase, insulin resistance of peripheral tissues increases, while liponeogenesis is reduced. Cancer patients also have problems with inadequate food intake, reduced ability to digest and assimilate food, and disturbed body homeostasis. The role of nutrition in cancer therapy should be emphasized because cancer, together with the long-term and intensive treatment process, may involve a high risk of malnutrition and other nutritional deficiencies in oncological patients. Malnutrition in patients can have a significant impact on the immune system, driving worse treatment outcomes and clinical remission [7]. When irreversible refractory cachexia develops in a patient with colorectal cancer, life expectancy is <3 months. There are few oncological treatment options for such a patient, and a very poor response to anti-cancer treatment is common. Catabolism progresses at an accelerating rate and has no effect on nutritional support [8]. In a study by Baracos et al,. an increase in weight loss depending on the primary location of the tumor was found in approximately 45% of colorectal cancer patients. The average weight loss is about 7 kg in patients with colorectal cancer, the risk of developing cancer cachexia is high in this group of patients [9].

Malnutrition in an oncology patient leads to a general weakening of the body, reduces the action of the body’s defense forces (reduces immunity), affects the prolonged recovery period and leads to lower tolerance of the side effects of cancer therapy. In addition, malnutrition increases treatment toxicity, reduces quality of life and accounts for 10% to 20% of deaths in cancer patients [10]. Patients with diagnosed and treated colorectal cancer are at higher risk of malnutrition; studies show that significant weight loss is observed in patients with advanced stage gastrointestinal cancers (gastric, pancreatic, colorectal cancer) [11]. The nutrition of CRC patients is hampered by the lack of an agreed position on the method for assessing the nutritional status of cancer patients. ESPEN recommends the use of Nutritional Risk Screening (NRS). The study by Thoresen et al. demonstrated that Subjective Global Assessment (SGA) is a valuable method of assessing the nutritional status of patients with advanced cancer, as is the patient-generated SGA modified by Persson et al. [12]. For this reason, knowledge in the field of nutrition of oncology patients requires further research and expansion.

This review aimed to describe the available data about clinical nutrition in colorectal cancer patients for future research in this area. In the article we described the prevalence of colorectal cancer, treatment methods and their side effects related to the nutrition state of the patient, and the role of individual food components in the process of patient therapy.

## 2. Materials and Methods

In the first quarter of 2022, a general literature review was carried out to summarize key aspects related to nutrition in colorectal cancer. The following searching strategy was applied: articles were extracted from PubMed and grey literature using the terms “clinical nutrition”, “nutrition”, and in combination with “colorectal cancer”. The most relevant and essential literature in the field was identified using expert knowledge on nutrition in CRC and incorporated into the article.

## 3. Nutrition Treatment of Patients with Colorectal Cancer

### 3.1. Epidemiology and Risk Factors

Colorectal cancer is one of the most common malignant neoplasms in the world and is the third leading cause of cancer death in the world. According to data from World Cancer Research Fund International, in 2020 over 1.9 million new cases of colorectal cancer were registered in total. In 2020 there were 935,000 deaths due to cancer in the world. The highest risk of CRC is observed in developed countries in Europe, Australia and North America. According to the WCRFI, Poland was in 7th place with 15,088 deaths in terms of the standardized ASR 16.1/100,000. In terms of gender, Poland came 6th for men (ASR = 22.8/100,000) and 10th for women (ASR = 11.3/100,000). Colorectal cancer has a higher incidence among men than women and age is a major risk factor for this cancer. The incidence increases between 40 to 50 [13].

The prevalence of colorectal cancer in various regions of the world is different, partly due to the genetic predisposition of a given population, but environmental factors may also be responsible for the different incidence rates, which depend on the specificity of a given area and the lifestyle of the people living there [14,15,16]. The risk of colorectal cancer increases approximately linearly with increasing body mass index (BMI) between 23–30 kg/m^2^. Patients with a BMI > 30 kg/m^2^ have a 50–100% increased risk compared with those with a BMI < 23 kg/m^2^. This relation is stronger in men than in women. The International Agency for Research on Cancer (IARC) has classified red meat as Group 2A: substances probably carcinogenic to humans. A prospective cohort study was conducted in the UK to determine the association between red meat consumption and the incidence of the 20 most common cancers. The study enrolled 474,996 people aged 37–73 years with no history of cancer. The observation period was 7 years. A malignant tumor was diagnosed in 28,955 participants. Consumption of red and processed meat was significantly associated with a higher risk of developing colorectal cancer. It was estimated that for every 100 grams of red meat consumed daily, the risk of colorectal cancer increased by 17% [17].

### 3.2. Treatment and Side Effects

The basic treatment of colorectal cancer is a classical or laparoscopic resection of the tumor with the removal of the surrounding lymph nodes. Radical radiotherapy may be an alternative in selected rectal cancer patients. In addition, systemic chemotherapeutic treatment is used with drugs such as 5-fluorouracil biomodulated with folinic acid, irinotecan, oxaliplatin, and also molecularly targeted drugs such as bevacizumab, cetuximab and panitumumab, etc. Symptomatic treatment of patients with colorectal cancer involves keeping the digestive tract unobstructed and controlling bleeding using surgical and/or endoscopic techniques. Surgical procedures that result in a stoma are nowadays rare, mostly related to the treatment of colorectal cancer in the rectum below the peritoneal refraction where the tumor is close to the rectal sphincter or invades the sphincter [3,15].

The period after primary oncological treatment is the next phase of cancer therapy, such as in colorectal cancer [18]. The aims of care during this time should be control of relapses, ongoing supervision of late and long-term side effects of anticancer treatment, as well as secondary prevention, including supporting the implementation of healthy lifestyle principles. Due to the fact that most cases of colorectal cancer are formed from polyps (benign adenocarcinomas) in the lumen of the large intestine, and the fact that their transformation into malignant cancer can take up to 10 years without showing specific symptoms [3], secondary prevention must be carried out continuously and under the control of specialists to ensure proper implementation and adherence to its principles [19].

The most common side effects of anticancer treatment for colorectal cancer, which may persist even after the end of oncological treatment, are neuropathy caused by the use of oxaliplatin and gastrointestinal disorders, which occur as a result of pelvic radiotherapy and the use of chemotherapeutics (Table 1 and Table 2). Although chemotherapeutics mainly reduce cell division in its different stages, ionizing rays cause overlapping damage to the epithelium, microstructure, intestinal nervous system and disorders of intestinal microbiota [20,21,22,23].

Delayed damage to the large intestine occurs about 3 months or more after radiotherapy and is characterized by mucosal atrophy, sclerosis of vessels and progressive fibrosis of the intestinal wall. The symptoms are chronic, progressive and mainly characterized by poor nutrient absorption and abnormal transport of intestinal contents [20]. In addition, the acute toxicity of radiotherapy is associated with increased inflammation, while chronic toxicity is associated with fibrosis and sclerosis of blood vessels located in the intestinal wall. Intestinal disorders may be of differing severity and forms, depending on the choice of therapy method, length of use, as well as the individual patient’s predisposition or decision to include nutritional treatment [18]. Chronic diarrhea occurs in about 25% of people in remission, and for 13–50% of those people, this symptom can be found up to 10 years after the end of treatment [24]. Among those in remission who have undergone intestinal resection, intestinal disorders are the most problematic in the first year after surgery and are also associated with a lower quality of life. The use of radiotherapy, both before and after the procedure, additionally increases the risk of long-term changes in the functioning of the gastrointestinal tract, such as peristalsis disorders and the inability to cease defecating [25]. Peripheral neuropathy, induced by the dose-dependent toxicity of the chemotherapeutic drug oxaliplatin, manifests itself in sensory disturbances in peripheral nerves and the presence of gloves-and-socks syndrome. The symptoms are mainly pain, paraesthesia, numbness, sensory disturbances and a change in proprioceptive feeling, which limit the ability to move and perform activities such as dressing, preparing meals, holding objects (including cutlery) and writing [24].

### 3.3. Nutrition in Cancer Therapy

The principles of nutrition during anticancer therapy should mainly consider light and low-fat foods, the exclusion of lactose and gluten-containing foods in certain cases or the introduction of special dietary products such as oral nutrition supplements. Recommended individual nutritional intervention that focuses on increased nutrient demand in its principles and the mitigation of late treatment side effects observed in some colorectal cancer survivors may have beneficial effects (Table A1 in Appendix A) [26,27,28].

Cancer is inseparable from the reduction or exhaustion of body protein reserves and many vitamins and minerals [28]. The reserves are mobilized by intensifying selected metabolic pathways: anaerobic glycolysis, gluconeogenesis and lipolysis are intensified, insulin resistance of peripheral tissues increases, while the process of liponeogenesis is limited. In the meantime, the protein metabolism intensifies the proteolysis of skeletal muscle proteins. These changes are aimed at improving the easily accessible pool of energy substrates (glucose) and building blocks in the body, mainly used for the current synthesis of immune system elements: antibodies, antioxidant enzymes and proteins involved in inflammatory reactions, and for the synthesis of cancer proteins [28]. Among the nutrients most lost by colorectal cancer patients, the following should be mentioned: vitamin D, selenium, zinc, iron, vitamin C, vitamin E, folic acid and electrolytes [4,5,6,29]. For this reason, as well as due to insufficient food intake, reduced ability to digest and absorb food and disturbed homeostasis of the body (inflammation, mobilization of body reserves for energy purposes or the predominance of catabolic over anabolic reactions), patients with cancer rapidly develop numerous nutritional deficiencies. Once remission has been achieved, it could potentially last a long time as consumption of a traditional diet could be insufficient to meet the current nutritional demand, and even less sufficient to replenish the depleted body reserves. The restoration of these reserves is significant because of the greater risk of developing further cancerous changes than in other people [30].

The energy requirements of a colorectal cancer patient should be assessed individually. This can be done using indirect calorimetry. This method is currently considered the gold standard, but its application generates constant costs and requires appropriately trained personnel. Indirect calorimetry is currently the most precise method of determining the energy demand in hospital conditions. However, the lack of appropriate equipment in clinical wards forces the use of formulas. One of them is the formula developed by Harris and Benedict. According to the recommendations of the European Society for Parenteral and Enteral Nutrition (ESPEN), in the case of inability to determine resting energy requirements (REE) on an individual level, it may be assumed that a cancer patient requires 25–30 kcal/kg of current body weight per day. Malnourished patients should have increased demand, obese patients, a decreased demand [10].

There is still a lack of more individualized methods to assess demand, taking into account all the components that make up the total energy requirements of total energy expenditure (TEE). Most studies refer to basal metabolic REE, not including physical activity level (PAL) or postprandial thermogenesis.

According to studies, 2/3 of cancer patients take <25 kcal/kg body weight per day. This means that they do not cover the minimum requirements of the body, not to mention the needs resulting from anabolic processes associated with recovery [8].

Determining the individual protein, fat and carbohydrate requirements of patients with colorectal cancer is not straightforward. ESPEN recommendations in this respect indicate an increased need for protein. A patient with cancer should consume >1 g of protein per kilogram of body weight per day. It is advisable to aim for a supply of 1.5 g/kg body weight per day. The average non-protein energy intake should be 130 kcal/1 g nitrogen. To increase protein supply > 1.5 g/kg body weight per day, renal function should be monitored. In progressive renal failure with increasing creatinine, increasing protein in the diet should be done with caution [10]. ESPEN recommendations indicate that in patients with advanced colorectal cancer undergoing chemotherapy who are at risk of weight loss or malnourished, supplementation with long-chain omega-3 fatty acids or fish oil may be considered to stabilize or increase appetite and increase lean body mass. Current ESPEN recommendations indicate that it is not necessary to fortify special amino acids in clinical feeding mixes. The recommended supply of vitamins and minerals is approximately equal to the recommended daily allowance.

If the patient requires treatment with radiotherapy, in order to limit the occurrence of side effects during treatment called radiation reactions, it is important to follow the dietary recommendations during the treatment. A diet that is easy to digest and excludes foods that are hard to digest, fatty foods, raw milk, raw vegetables and fruits, fizzy drinks, juices and spices (especially important in the case of irradiation of the abdomen and pelvic region). Restriction of fiber and lactose is sometimes advisable in order to reduce such side effects as the occurrence of bloating, abdominal pain and diarrhea. Increased protein intake is also advisable (sources: low-fat cottage cheese, fermented milk products, lean poultry meat, rabbit meat, sea fish, small amounts of eggs, tofu cheese). The number of vegetables consumed should be increased, given in the form in which the patient will tolerate them—cooked, pureed. Strongly salted, pickled, smoked, fried products, alcohol and natural coffee should be eliminated from the diet [10].

### 3.4. Supportive Therapy after Surgical Resection

Several factors, such as dietary changes, increased physical activity, vitamin D use and coffee intake, have been shown to be beneficial in the setting after colorectal cancer surgery. Although there are no randomized clinical trials that have evaluated the role of diet after surgery, at least two studies have shown that patients who were on a diet with increased processed meat, red meat, sweets and refined grains had increased recurrence rates and decreased disease-free survival (DFS) [31,32,33]. High glycemic index diets were also associated with decreased DFS among obese and overweight patients [33,34].

Coffee consumption has been shown to reduce mortality risk, even after accounting for other potential confounders such as glycemic index, physical activity and other dietary factors [35]. In another observational study, higher coffee consumption in patients with stage III colon cancer was associated with reduced CRC-specific and all-cause mortality [36]. Each cup of coffee translated into an 18% lower rate of CRC-specific mortality and a 20% lower rate of mortality from any cause [37]. In another observational study, patients who increased their dietary fiber intake after being diagnosed with colorectal cancer had lower CRC-specific and all-cause mortality (19% and 14% lower risk per 5 g/day increase, respectively) [37].

Oral nutritional supplements (ONS), which are characterized by convenient and ready-made advantages, are widely considered a preferred nutritional intervention in patients who are at risk of malnutrition. The use of ONS developed for special medical purposes can increase nutrients and energy through the oral route to complement insufficient regular food intake [38,39]. Numerous studies have shown the beneficial impacts of ONS on nutritional status and clinical outcomes [40,41,42,43,44]. In addition to these beneficial effects, ONS treatment can lead to overall medical cost savings; it is also cost-effective [9,45].

A recently published a randomized clinical trial showed that the use of ONS can reduce skeletal muscle loss and the incidence of sarcopenia, and further improve chemotherapy tolerance in post-hospital discharge patients with nutritional risk after colorectal cancer surgery. These findings highlight the importance of ONS treatment in post-hospital discharge patients at nutritional risk after colorectal cancer surgery [45].

As a consequence of surgery, due to colorectal cancer resection, a fragment of the gastrointestinal tract is damaged, while the use of chemotherapeutics and radiotherapy is associated with damage to the gastrointestinal mucous membrane, e.g., in the form of mucositis, i.e., transient mucositis [46]. These situations require the intensification of processes related to tissue regeneration, which require, among other things, higher protein content in the diet, including exogenous amino acid glutamine, for the proper course. This component is important for rapidly dividing cells and is one of the main reservoirs of nitrogen for the body. In the case of oncological patients, its importance for the proper functioning of immune cells and the intestinal epithelium is proven [47]. The consequence of not providing the necessary nutrients within the diet is that they are drawn from the body’s reserves, which, in a situation of shortages, the risk of which is high after a period of treatment, may significantly reduce the healing process of the gastrointestinal mucosa, as well as the current immune defense, especially within the gastrointestinal tract, including the GALT immune system associated with the intestinal mucosa (Gut-Associated Lymphoid Tissue).

To reduce gastrointestinal disorders, it is also important to overturn the normal intestinal microbiota, which, provided it has the correct structure and activity, has a positive effect on the functioning of both the gastrointestinal tract and the GALT system located in it. This system is the body’s first line of defense against pathogens and toxins eaten with food. Its role is, among other things, to distinguish between commensal and pathogenic bacteria and to adjust the reaction depending on the diagnosis. When the intestinal microbiota is disturbed, as a result of, e.g., antibiotics, ionizing radiation, prolonged severe stress or a poor diet (rich in fats and monosaccharides) [48,49,50], the consequences may be a loss of intestinal barrier tightness, reduced production of B vitamins and anti-inflammatory substances (e.g., SCFA, Short-Chain Fatty Acids) and intensification of pro-inflammatory ones, e.g., LPS (Lipopolysaccharide) endotoxins [49,51,52]. As a result, these changes may lead to an increase in chronic inflammation, endotoxemia, an increase in the risk of infection and an increase in bloating and peristalsis disorders.

The nutrient that positively affects both the activity of normal intestinal microbiota and regulates the functioning of the digestive tract is dietary fiber. It is a set of ingredients of different structures, which do not undergo digestion under the influence of gastrointestinal enzymes. The general effect of dietary fiber intake is to increase fecal masses, stimulate intestinal peristalsis, slow down the absorption of nutrients and provide bacteria with substrates for energy production. However, various types of fiber (soluble, insoluble, prebiotics) may have different effects. To support the intestinal microbiota, it is best to take prebiotics that selectively support the growth and activity of only certain bacteria, especially Bifidobacterium and Lactobacillus. Prebiotics, in the course of lactic fermentation carried out by bacteria, become a source of energy for them, during which also such compounds as lactic acid, SCFA, hydrogen, methane and carbon dioxide are formed [53]. Compounds showing prebiotic features include fructooligosaccharides (FOS), galacto-oligosaccharides (GOS), xylooligosaccharides (XOS), isomaltooligosaccharides (IMO), soybean oligosaccharides (SBOS), lactulose, resistant starch and inulin [54]. The consequences of prebiotic administration may include, among others, more intense expression of tight mucosal conjugate proteins, a decrease in the total concentration of LPS bacterial endotoxin as well as a decrease in the generalized inflammation within the gastrointestinal tract [55,56].

Some patients will require nutritional intervention (enteral or parenteral nutrition) during, before, or after treatment. According to ESPEN recommendations, nutritional intervention is recommended in patients:

- with restricted food intake (less than 60% of daily requirements) for more than 10 days,
- with normal nutritional status, in whom a period of non-eating for at least 7 days is expected,
- meeting the criteria for malnutrition at the time of the treatment.

A thorough assessment of the patient’s clinical condition supplemented with nutritional history (changes in eating habits, symptoms of intolerance: lack of appetite, nausea, vomiting, diarrhea, constipation, etc.), analysis of the state and degree of malnutrition, treatment plan including nutritional therapy, type of treatment (surgery, chemotherapy, radiotherapy) allow for the selection of the method of management.

The answer to the following questions is crucial in the choice of the method and route of treatment: Is it possible to nourish a patient with colorectal cancer by the gastrointestinal route, allowing for full or partial satisfaction of the due daily protein-calorie requirements? Are there any contraindications to oral nutrition? Oral feeding is the most physiological way of nourishing the patient and if there are no contraindications, we should use the gastrointestinal route.

It is well known that when artificial nutrition techniques are used in oncological surgery, such management reduces the rate of postoperative complications and mortality. In the case of indications for preoperative nutritional support in patients with colorectal cancer, the optimal duration of nutritional support is 10–14 days, whereas in the postoperative period, as long as the clinical situation justifies it and until the patient starts oral nutrition, providing at least 60% of energy requirements [8].

Inability to feed by the oral route requires consideration of enteral nutrition (EN). Enteral feeding is indicated when a patient cannot eat normally or eats in insufficient amounts because of the following disorders: swallowing, chewing and salivary secretion (head and neck cancer), neurological (primary tumors and tumors metastatic to the central nervous system) and digestive system (toxicity of oncological treatment, postoperative states). In the case of enteral feeding, the principle of diet supply to the most efficient place in the gastrointestinal tract is applied. Initially, intragastric and later enteral access is considered.

Gastric access is achieved by inserting a nasogastric tube through the nose (this also applies to cancer patients with minor upper gastrointestinal lesions if the duration of the planned intervention is less than 30 days). This is followed by the establishment of a nutritional fistula using endoscopic or surgical methods, especially in patients with planned long-term or definitive access. The gold standard for this treatment is percutaneous endoscopic gastrostomy (PEG). Gastric lesions, gastric emptying disorders and the use of the stomach for gastrointestinal reconstructive procedures are indications for the delivery of an industrial diet to the small intestine. In intragastric nutrition (by gavage or gastrostomy) the following methods of diet delivery are most often used: a single portion of 200–300 mL given 5–6 times a day, a single portion of 50–100 mL several times a day, to cover daily requirements, continuous infusion and delivery by gravity system in the form of a drip infusion or by means of a volumetric pump.

According to ESPEN guidelines, parenteral nutrition is recommended in cancer patients to improve their function and treatment outcomes. Parenteral nutrition is recommended in case of complete lack of possibility of industrial diet delivery into the gastrointestinal tract or as a supplement in so-called mixed nutrition, which is a more than 40% supplement of daily protein and caloric needs. Parenteral nutrition is usually carried out using central access through cannulation of large venous vessels of the superior vena cava outflow. This type of access allows long-term feeding, or peripheral access, through cannulation of peripheral veins, mainly in the forearms, with short cannulas. This type of access is reserved for a feeding period not longer than 7–14 days with the need to use fat emulsion in the mixture. In the study by Costanzo et al. [57], it was proved that parenteral nutrition prolongs survival time in patients with cancer and led to statistically significantly longer survival by 3 months in malnourished patients with disseminated disease [57].

## 4. Conclusions

Malnutrition negatively impacts the quality of life and increases treatment toxicities, and it has been estimated that up to 10–20% of cancer patients die due to the consequences of malnutrition rather than because of the tumor itself. Thus, nutrition plays a crucial role in multimodal cancer care. Robust evidence indicates that nutritional issues should be taken into account from the time of cancer diagnosis, within a diagnostic and therapeutic pathway, and should be running in parallel to antineoplastic treatments. However, worldwide, cancer-related malnutrition is still largely unrecognized, underestimated and undertreated in clinical practice [10].

Each patient should have the opportunity to receive dietary consultation and development of a nutritional plan for the period of treatment and after the completion of treatment by a qualified dietitian specializing in clinical nutrition in oncology to minimize the risk of malnutrition and other complications that, as indicated by the above studies, affect the prognosis and quality of life of the cancer patient. In a study by Muscaritoli et al. [58] among 1952 cancer patients at their first visit to an oncologist, 51% were diagnosed with malnutrition, 9% were obese, 43% were at high nutritional risk and 40% of patients were anorexic [58]. In Poland, many oncology departments do not have a dietician and the patient can only benefit from a paid, private visit.

The use of ONS may reduce skeletal muscle loss and the incidence of sarcopenia, and further improve chemotherapy tolerance in patients after hospital discharge with nutritional risk after colorectal cancer surgery. Increasing dietary fiber intake after a colorectal cancer diagnosis have lower specific mortality [37].

In order to promote the body’s compensatory capacity to fight cancer and to secure a reserve of body nutrients to prevent the development of malnutrition, a regular and uninterrupted supply of particular nutrients is necessary at every stage of cancer treatment for colorectal cancer and after its completion. Due to the treatment being carried out at the limit of the body’s tolerance, with the risk of serious side effects of the treatment and limited possibilities of consuming a customary diet, an additional supply of several nutrients is desirable.

These are mainly substrates participating in the regenerative processes, and nutrients necessary for the proper functioning of the immune system, including the reconstruction of the GALT system and the efficient course of antioxidant mechanisms.

This group of nutrients also includes substances participating in the healing of the intestinal mucosa and its nervous tissue [4].

Ultimately, however, further randomized controlled trials on the intake of individual macronutrients and micronutrients from the diet and the gut microbiome of colorectal cancer patients are needed; these trials may play a prognostic role and influence treatment outcome and prognosis in this group of patients.

## Figures and Tables

**Table 1:** Side effects of chemotherapeutics with negative effects on the nutrition status [20,21,22,23].

Group of Medicines	Examples of Medicines	Possible Side Effects Affecting the Nutrition Status
pyrimidine analogues	5-fluorouracil, capecitabine,	nausea, vomiting, neutropenia the frequency of mucositis increases with the bolus dose, the frequency of cycles, diarrhea
platinum analogue	oxaliplatin	Often: Nausea, vomiting, abdominal pain, constipation, peripheral neuropathy
topoisomerase II inhibitors	irinotecan	neutropenia Often: dehydration due to diarrhea and/or constipation, infections, acute cholinergic syndrome Rare: pseudomembranous gastroenteritis, intestinal obstruction or gastrointestinal bleeding, neutropenia

**Table 2:** Side effects of biological drugs (recombinant monoclonal bodies) with can have a negative impact on the nutrition status [20,21,22,23].

Medicine	Possible Side Effects to the Nutrition Status or Dietary Modifications
bevacizumab	hypertension, rectal bleeding, impeded wound healing, proteinuria
cetuximab	infusion allergic reactions, diarrhea, nausea, vomiting, dehydration (especially following diarrhea or mucositis), electrolyte disturbances, hypocalcemia, hypomagnesemia, hypokalemia), skin rash, acne rash, dry skin, nail changes, weakness, weight loss, skin infections, respiratory infections
panitumumab	diarrhea, nausea, vomiting, hypokalemia, hypomagnesemia, weight loss, skin rash, acne rash, dry skin, nail changes, weakness

## Data Availability

Not applicable.

## Appendix A

### Regenerative Mixture for Patients during the Treatment and after Achieving Remission of Colorectal Cancer

Indications for use: As a supplement to the diet of patients with colorectal cancer during treatment (between chemotherapy courses) and after first-line treatment, to reduce nutrient deficiencies, strengthen immune system functions, and stimulate regeneration processes.

**Table A1:** Mixture composition (quantity per day).

**Colorectal Cancer**
daily portion
**Water-soluble vitamins**
B1 10 mg
B3 (niacin) 3 mg
B2 4 mg
B6 4 mg
B12 29 µg
B5 (Dexpanthenol) 500 mg
Folic acid 400 µg
C 250 mg
**Fat-soluble vitamins**
A 130 µg of beta-carotene
D3 4000 µg
E all-rac-α-tocoferol 6.67 µg
K2 1.42 mg
**Trace elements**
Zinc (Zn) 30 mg (in the form of gluconate zinc)
Selenium (Se) 500 µg (in the form of selenium sodium)
Magnesium (Mg) 64 ions—as citrate or other organic compound)
Calcium (Ca) 300 mg as calcium citrate (not calcium carbonate)
**Total carbohydrates 20 g**
maltodextrin 60%
maltose 20%
glucose 20%
**Protein 23.5 g, including amino acids:**
Isoleucine 1 g
Leucine 1.3 g
Valine 1 g
Lysine 0.9 g
L Phenylalanine 0.09 g
Methionine 0.1 g
L-cysteine 0.05 g
L-threonine 0.4 g
L-tryptophan 0.07 g
L-arginine 1.0 g
L-histitidine 0.3 g
Glycine 0.58 g
L-alanine 0.46 g
L-proline 057 g
L-serine 0.22 g
**Fats 4 g**
Omega 3 polyunsaturated fatty acids (EPA and DEH), 2 g Medium-chain triglycerides MCT 2 g
**Additives dedicated for colorectal cancer patients:**
SCFA butyric acid 1 g (tributyrin)
glutamin 30 g
nucleotide acids 1.29 (yeast extract)
arginine 1.0 g
choline 1 g
iron 8 mg (iron bicarbonate or fumarate)
Water-soluble dietary fiber (prebiotic) Inulin 2 g Fructooligosaccharides 3 g
**Soy bean lecithin—as a stabilizer of the water and fat fraction (depending on the mixture stability)**
